# Mortality trends in an ambulatory multidisciplinary heart failure unit from 2001 to 2018

**DOI:** 10.1038/s41598-020-79926-3

**Published:** 2021-01-12

**Authors:** Giosafat Spitaleri, Josep Lupón, Mar Domingo, Evelyn Santiago-Vacas, Pau Codina, Elisabet Zamora, Germán Cediel, Javier Santesmases, Crisanto Diez-Quevedo, Maria Isabel Troya, Maria Boldo, Salvador Altmir, Nuria Alonso, Beatriz González, Julio Núñez, Antoni Bayes-Genis

**Affiliations:** 1 Heart Failure Clinic and Cardiology Service, University Hospital Germans Trias I Pujol, Badalona, Spain; 2 Department of Medicine, Universitat Autonoma de Barcelona, Barcelona, Spain; 3 CIBERCV, Instituto de Salud Carlos III, Madrid, Spain; 4 Cardiology Department, Hospital Clínico Universitario, INCLIVA, València, Spain; 5 Department of Medicine, Universitat de València, València, Spain; 6 Heart Institute, Hospital Universitari Germans Trias I Pujol, Department of Medicine, Universitat Autonoma de Barcelona, Carretera del Canyet s/n 08916, Badalona, Spain

**Keywords:** Cardiology, Heart failure

## Abstract

To assess mortality trends at 1 and 3 years from 2001 to 2018 in a real-life cohort of HF outpatients from different etiologies with depressed and preserved LVEF. A total of 2368 consecutive patients with HF (mean age 66.4 ± 12.9 years, 71% men, 15.4% with preserved LVEF) admitted to a HF clinic from August 2001 to September 2018 were included in the study. Patients were divided into five quintiles (Q) according to the period of admission. Trends for all-cause and cardiovascular mortality from Q1 to Q5 were assessed by linear regression. Patients with LVEF < 50% had a progressive decrease in the rates of all-cause and cardiovascular death at 1 year (12.1% in Q1 to 6.5% in Q5, p = 0.003; and 8.4% in Q1 to 3.8% in Q5, p = 0.007, respectively) and 3 years (30.5% in Q1 to 17.0% in Q5, p = 0.003; and 23.9% in Q1 to 9.8% in Q5, p = 0.003, respectively). These trends remained significant after adjusting for clinical characteristics and risk. No significant trend in mortality was observed in patients with LVEF ≥ 50%. In a cohort of real-life ambulatory patients with HF, mortality progressively declined in patients with LVEF < 50%, but the same trend was not observed in patients with preserved LVEF.

## Introduction

Over the last three decades, since the introduction of angiotensin-converting enzyme inhibitors (ACEIs), there has been a progressive reduction in mortality rates across trials testing new therapies in patients with heart failure (HF) and reduced left ventricular ejection fraction (LVEF)^1–10^. Therefore, current guidelines recommend the use of drugs inhibiting the neurohormonal axis and devices, such as implantable cardioverter defibrillator (ICD) and cardiac resynchronization therapy (CRT), to prolong survival in patients with HF and reduced LVEF (HFrEF)^11^. Despite advances in HF management, which also include the implementation of HF clinics and specialized HF teams, the prognosis is still poor in the real-life setting. Data from registries indicate that, regardless of ejection fraction, mortality in HF patients can be as high as 75% at 5 years^12^, a rate that is significantly higher than in clinical trials, in which patients are usually younger and have fewer comorbidities. In addition, no improvement in survival has been observed across trials enrolling patients with HF with preserved LVEF (HFpEF). Furthermore, despite encouraging results from recent studies, no convincing evidence-based therapy for HFpEF has been identified^13,14^. Thus, mortality from HF remains a challenge in real-world practice.

In the present study, we assessed mortality trends at 1 and 3 years, from 2001 to 2018, in a real-life cohort of HF outpatients with depressed and preserved LVEF.

## Methods

### Study population and outcomes

All consecutive ambulatory patients admitted to a structured multidisciplinary HF clinic at a university hospital between August 2001 and September 2018, regardless of aetiology, were considered for the study. During the 19-year study period, the clinical pathways and referral geographic area, covering ~ 850,000 inhabitants in the northern Barcelona Metro Area, remained stable. Patients were referred to the HF clinic mostly by the Cardiology or Internal Medicine Departments, and to a lesser extent by the Emergency Department or other hospital departments. The criteria for referral to the HF clinic were HF according to the European Society of Cardiology (ESC) guidelines, regardless of aetiology, at least one HF hospitalization, and/or reduced LVEF^15,16^. All patients were seen regularly for follow-up visits at the HF clinic according to their clinical needs and treated according to a unified protocol. Follow-up visits included a minimum of one visit with a nurse every 3 months and one visit with a physician (cardiologist, internist, or family physician) every 6 months, as well as optional visits with specialists in geriatrics, psychiatry, and rehabilitation^15,16^, with the addition of a nephrologist and endocrinologist in recent years.

Patients were divided into quintiles according to the period of admission as follows: quintile 1 (Q1) from August 3, 2001, to August 31, 2004; quintile 2 (Q2) from September 1, 2004, to November 10, 2008; quintile 3 (Q3) from November 11, 2008, to June 22, 2012; quintile 4 (Q4) from June 23, 2012, to October 9, 2015; quintile 5 (Q5) from October 10, 2015, to September 28, 2018. For the purpose of this study, all-cause and cardiovascular death rates were estimated 1 and 3 years after admission in all quintiles. In addition, recurrent HF-related hospitalizations during the same study time periods were assessed. In order to avoid possible bias due to different follow-up times, we calculated the number of HF hospitalizations per 100 patients-years for patients of each admission period. Mortality trends were assessed across the study period in the overall population and in the depressed (a category that includes patients with LVEF < 50%) and preserved (LVEF ≥ 50%) subgroups. Fatal events were identified from the patients’ health records (including hospital wards, emergency room, and general practitioners) or by contacting their relatives. Data were verified using the databases of the Catalan and Spanish Health Systems. Events were adjudicated by an ad hoc committee (JL, MdeA, BG, and MD; PM resolved the possible discrepancies). No loss of follow-up at 5 years was observed for vital status. For HF-related hospitalizations, 37 patients were lost for hospitalizations data during follow-up due to geographic mobility. These patients were adequately censored at the time of transfer. During the baseline visit, patients provided written consent for the use of their clinical data for research purposes. The study was performed in compliance with the law protecting personal data in accordance with the international guidelines on clinical investigations from the World Medical Association’s Declaration of Helsinki. Informed consent was obtained from all participants. The local ethics committee (Research Ethics Committee, Germans Trias i Pujol University Hospital) approved the study (ethic code REGI-UNIC PI-18–037).

### Statistical analysis

Categorical variables were expressed as absolute numbers and percentages. Continuous variables were expressed as the mean ± standard deviation (SD) or median [interquartile range] according to normal or non-normal distributions. Normal distributions were assessed by normal Q to Q plots. Trends in baseline characteristics across quintiles were assessed by the Mantel–Haenszel test of trend for categorical variables and linear regression for continuous variables.

Patients with depressed and preserved LVEF were compared using chi-squared and Fisher’s exact test for categorical variables, and the Student’s t-test or U Mann–Whitney test for continuous variables, as appropriate. Trends for all-cause and cardiovascular mortality from the first to the last quintile were assessed by linear regression, using the period of admission as the independent variable. To analyse the potential confounding effect of baseline variables on the risk of all-cause and cardiovascular death, two multiple regression models were used. In model 1, stepwise multiple regression analyses, including age, sex, LVEF, NYHA class III-IV, and number of comorbidities (among diabetes, hypertension, anaemia, atrial fibrillation or flutter, chronic obstructive pulmonary disease [COPD]) as covariates with quintile of admission, were performed. In model 2, quintile of admission and Meta-Analysis Global Group in Chronic (MAGGIC) Heart Failure Risk Score (at 1 or 3 years) were included and analysed using standard multiple regression. Time to event curves are presented by quintiles of the period of admission as estimated using the Kaplan–Meier method. Statistical analyses were performed in SPSS 24 (SPSS Inc., Chicago, IL, USA). A two-sided P < 0.05 was considered significant.

## Results

### Baseline characteristics

From August 2001 to September 2018, 2368 patients were admitted to our HF clinic. Of these, 2004 (84.6%) presented with depressed LVEF and 364 (15.4%) with preserved LVEF at admission. Table 1 summarizes the baseline demographic and clinical characteristics of patients and treatments during follow-up, showing the differences between the two LVEF groups. Overall, the mean age was 66.4 ± 12.9 years, 71% were males, and the prevalence of ischemic heart disease was 48%. Table 2 shows the data according to period of admission quintiles and LVEF subgroups. Regardless of the LVEF, patients admitted in the latter periods reported less severe NYHA functional class symptoms. In the depressed LVEF subgroup, patients admitted more recently had shorter HF duration, higher LVEF, lower 1- and 3-year death risk by MAGGIC score, but more comorbidities. Conversely, patients with LVEF ≥ 50% were progressively younger, more often female, and had fewer comorbidities.

**Table 1:** Baseline demographic and clinical characteristics in patients with depressed and preserved ejection fraction (EF).

	Total cohort (n = 2368)	Depressed EF (n = 2004)	Preserved EF (n = 364)	p-value
Age, years	66.4 ± 12.9	65.9 ± 12.5	69.1 ± 14.5	< 0.001
Male	1681 (71.0)	1505 (75.1)	176 (48.4)	< 0.001
Caucasian	2298 (97.0)	1941 (96.9)	357 (98.1)	0.42
**Aetiology**				< 0.001
Ischaemic heart disease	1141 (48.2)	1084 (54.1)	57 (15.7)	
Dilated CM	345 (14.6)	334 (16.7)	11 (3.0)	
Hypertensive CM	219 (9.2)	132 (6.6)	87 (23.9)	
Alcohol CM	115 (4.9)	111 (5.5)	4 (1.1)	
Drug-related CM	65 (2.7)	59 (2.9)	6 (1.6)	
Valvular	215 (9.1)	130 (6.5)	85 (23.4)	
Hypertrophic CM	72 (3.0)	6 (0.3)	66 (18.1)	
Amyloidosis	13 (0.5)	4 (0.2)	9 (2.5)	
Other	183 (7.7)	142 (7.2)	39 (10.7)	
HF duration, months	7 [1–44]	6 [1–40]	13 [3–57]	< 0.001
**NYHA class**				< 0.001
I	166 (7.0)	119 (5.9)	47 (12.9)	
II	1534 (64.8)	1356 (67.7)	178 (48.9)	
III	640 (27.0)	507 (25.3)	133 (36.5)	
IV	28 (1.2)	22 (1.1)	6 (1.6)	
LVEF, %	35.3 ± 14.2	30.5 ± 8.7	61.9 ± 7.8	< 0.001
LVEDD, mm*	59.7 ± 9.3	61.6 ± 8.2	48.8 ± 6.9	< 0.001
LVESD, mm^#^	47.0 ± 11.2	49.6 ± 9.6	32.4 ± 7.5	< 0.001
Δ LVEF at 1 year†	7.7 ± 12.0	8.8 ± 11.9	− 0.2 ± 9.8	< 0.001
Diabetes	1019 (43.0)	867 (43.3)	152 (41.8)	0.60
Hypertension	1505 (63.6)	1258 (62.8)	247 (67.9)	0.06
COPD	406 (17.1)	345 (17.2)	61 (16.8)	0.83
Anaemia^†,a^	1072 (45.4)	897 (44.9)	175 (48.2)	0.24
Renal insufficiency^§,b^	1035 (44.1)	850 (42.8)	185 (51.5)	0.002
Atrial fibrillation or flutter	511 (21.6)	360 (18.0)	151 (41.5)	< 0.001
Number of comorbidities	1.9 ± 1.2	1.8 ± 1.2	2.2 ± 1.3	< 0.001
1-year death risk (MAGGIC score)	13 [8–21]	13 ^8–21^	13[6–23]	0.10
3-year death risk (MAGGIC score)	32 [21–46]	32 [21–46]	32 [16–49]	0.0.10
BMI, kg/m^2‡^	27 [24–30]	26 [24–30]	27 [25–32]	0.03
Obesity^‡,c^	622 (26.5)	509 (25.6)	113 (31.4)	0.02
NT-proBNP, ng/L^¶^	1690 [711–4095]	1750 [764 -4263]	1300 [394–3210]	0.02
**Treatments**				
ACEI/ARB/ARNI	2040 (86.1)	1815 (90.6)	225 (61.8)	< 0.001
ARNI	257 (10.9)	252 (12.6)	5 (1.4)	< 0.001
Beta-blocker	2104 (88.9)	1842 (91.9)	262 (72.0)	< 0.001
MRA	1534 (64.8)	1366 (68.2)	168 (46.2)	< 0.001
Loop diuretic	2133 (90.1)	1836 (91.6)	297 (81.6)	< 0.001
Digoxin	908 (38.3)	789 (39.4)	119 (32.7)	0.016
Ivabradine	487 (20.6)	471 (23.5)	16 (4.4)	< 0.001
CRT	255 (10.8)	245 (12.2)	10 (2.7)	< 0.001
ICD	356 (15.0)	334 (16.7)	22 (6.0)	< 0.001

Data are given as mean ± standard deviation, median [interquartile range], or n (%).

*n = 2119; ^#^n = 2083; ^†^n = 1506; ^§^n = 2361; ^‡^n = 2345; ^¶^n = 2350; ^¥^n = 1665.

ACEI, angiotensin-converting enzyme inhibitor; ARB, angiotensin II receptor blocker; ARNI, angiotensin receptor–neprilysin inhibitor; BMI, body mass index; CM, cardiomyopathy; COPD, chronic obstructive pulmonary disease; CRT, cardiac resynchronization therapy; HF, heart failure; ICD, implantable cardiac defibrillator; MAGGIC, Meta-analysis Global Group in Chronic Heart Failure; MRA, mineralocorticoid receptor antagonist; NT-proBNP, N-terminal pro-brain natriuretic peptide; NYHA, New York Heart Association.

^a^According to World Health Organization criteria (< 13 g/dL in men and < 12 g/dL in women).

^b^Estimated glomerular filtration rate (Chronic Kidney Disease-Epidemiology Collaboration equation) < 60 mL/min/1.73 m^2^.

^c^Body mass index ≥ 30 kg/m^2^.

**Table 2:** Baseline characteristics based on period of admission quintiles and left ventricular ejection fraction group.

	Depressed LVEF (n = 2004)						Preserved LVEF (n = 364)					
	Quintile 1 (n = 423)	Quintile 2 (n = 412)	Quintile 3 (n = 417)	Quintile 4 (n = 385)	Quintile 5 (n = 367)	P for trend	Quintile 1 (n = 50)	Quintile 2 (n = 62)	Quintile 3 (n = 57)	Quintile 4 (n = 89)	Quintile 5 (n = 106)	P for trend
Age, years	65.5 ± 11.1	66.9 ± 12.6	66.3 ± 13.0	66.3 ± 12.7	64.5 ± 13.0	0.25	70.9 ± 9.9	70.5 ± 13.9	68.9 ± 16.1	72.4 ± 13.1	64.9 ± 16.0	0.03
Male	324 (76.6)	309 (75.0)	325 (77.9)	275 (71.4)	272 (74.1)	0.22	33 (66.0)	39 (62.9)	33 (57.9)	43 (48.3)	40 (37.7)	< 0.001
Caucasian	421 (99.5)	405 (98.3)	398 (95.4)	369 (95.8)	348 (94.8)	< 0.001	50 (100)	62 (100)	56 (98.2)	87 (97.8)	102 (96.2)	< 0.05
**Aetiology**						< 0.001						< 0.001
Ischaemic HD	271 (64.1)	245 (59.5)	229 (54.9)	185 (48.1)	154 (42.0)		11 (22.0)	12 (19.4)	7 (12.3)	13 (14.6)	14 (13.2)	
Dilated CM	49 (11.6)	38 (9.2)	76 (18.2)	73 (19.0)	98 (26.7)		1 (2.0)	0 (0.0)	2 (3.5)	4 (4.5)	4 (3.8)	
Hypertensive CM	33 (7.8)	30 (7.3)	21 (5.0)	31 (8.1)	17 (4.6)		15 (30.0)	17 (27.4)	17 (29.8)	24 (27.0)	14 (13.2)	
Alcohol CM	28 (6.6)	22 (5.3)	24 (5.8)	15 (3.9)	22 (6.0)		1 (2.0)	1 (1.6)	2 (3.5)	0 (0.0)	0 (0.0)	
Drug-related CM	6 (1.4)	10 (2.4)	14 (3.4)	17 (4.4)	12 (3.3)		1 (2.0)	1 (1.6)	1 (1.8)	2 (2.2)	1 (0.9)	
Valvular	17 (4.0)	39 (9.5)	23 (5.5)	30 (7.8)	21 (5.7)		16 (32.0)	23 (37.1)	14 (24.6)	20 (22.5)	12 (11.3)	
Hypertrophic CM	0 (0.0)	0 (0.0)	1 (0.2)	1 (0.3)	4 (1.1)		0 (0.0)	1 (1.6)	5 (8.8)	17 (19.1)	43 (40.6)	
Amyloidosis	0 (0.0)	0 (0.0)	0 (0.0)	0 (0.0)	4 (1.1)		0 (0.0)	0 (0.0)	4 (7.0)	3 (3.4)	2 (1.9)	
Other	19 (4.5)	28 (6.8)	29 (7.0)	33 (8.6)	35 (9.5)		5 (10.0)	7 (11.3)	5 (8.8)	6 (6.7)	16 (15.1)	
HF duration, months	24 [2–54]	4 [1–36]	6 [2–42]	6 [2–28]	3 [1–19]	< 0.001	14 [3–60]	20 [5–48]	14 [4–48]	7 [2–39]	23 [3–66]	0.99
**NYHA class**						< 0.001						< 0.001
I	17 (4.0)	33 (8.0)	12 (2.9)	9 (2.3)	48 (13.1)		1 (2.0)	3 (4.8)	5 (8.8)	8 (9.0)	30 (28.3)	
II	216 (51.1)	254 (61.7)	327 (78.4)	287 (74.5)	272 (74.1)		19 (38.0)	34 (54.8)	32 (56.1)	44 (49.4)	49 (46.2)	
III	175 (41.4)	121 (29.4)	77 (18.5)	87 (22.6)	47 (12.8)		27 (54.0)	23 (37.1)	20 (35.1)	36 (40.4)	27 (25.5)	
IV	15 (3.5)	4 (1.0)	1 (0.2)	2 (0.5)	0 (0.0)		3 (6.0)	2 (3.2)	0 (0.0)	1 (1.1)	0 (0.0)	
LVEF, %	29.2 ± 9.4	29.3 ± 8.5	30.3 ± 8.1	31.7 ± 8.4	32.3 ± 8.4	< 0.001	61.6 ± 8.6	61.3 ± 6.9	60.0 ± 7.8	62.7 ± 7.9	62.7 ± 7.8	0.14
LVEDD, mm*	62.2 ± 9.1	61.9 ± 8.3	61.8 ± 8.2	61.1 ± 7.8	61.1 ± 7.7	0.01	50.5 ± 7.3	49.5 ± 6.8	49.4 ± 8.1	49.1 ± 7.0	47.2 ± 5.8	0.03
LVESD, mm^#^	49.0 ± 10.5	49.7 ± 9.5	50.0 ± 9.4	49.9 ± 9.6	49.4 ± 9.1	0.52	32.9 ± 8.6	33.8 ± 6.8	34.1 ± 8.5	32.1 ± 7.6	31.0 ± 6.7	0.20
Δ LVEF at 1 year^†^	5.4 ± 11.0	7.8 ± 11.7	9.1 ± 11.4	10.5 ± 12.2	12.6 ± 12.4	0.001	− 2.4 ± 14.5	0.6 ± 9.6	− 0.6 ± 7.7	− 0.2 ± 8.1	0.7 ± 9.8	0.21
Diabetes	177 (41.8)	169 (41.0)	164 (39.3)	191 (49.6)	166 (45.2)	0.05	21 (42.0)	27 (43.5)	24 (42.1)	45 (50.6)	35 (33.0)	0.36
Hypertension	234 (55.3)	243 (59.0)	264 (63.3)	272 (70.6)	245 (66.8)	< 0.001	32 (64.0)	47 (75.8)	43 (75.4)	66 (74.2)	59 (55.7)	0.09
COPD	92 (21.7)	82 (19.9)	51 (12.2)	59 (15.3)	61(16.6)	0.01	9 (18.0)	14 (22.6)	8 (14.0)	13 (14.6)	17 (16.0)	0.41
Anaemia^§^^,a^	179 (42.3)	198 (48.3)	181 (43.4)	201 (52.6)	138 (37.7)	0.63	29 (58.0)	34 (54.8)	27 (47.4)	55 (62.5)	30 (28.3)	0.001
Renal insufficiency^‡^^,b^	160 (37.8)	161 (39.1)	169 (40.5)	212 (55.2)	148 (42.3)	0.001	22 (44.0)	28 (45.2)	32 (56.1)	65 (73.0)	38 (37.6)	0.86
AF/FT	55 (13.0)	70 (17.0)	98 (23.5)	75 (19.5)	62 (16.9)	0.07	28 (56.0)	29 (46.8)	25 (43.9)	38 (42.7)	31 (29.2)	0.002
Number of comorbidities	1.7 ± 1.1	1.8 ± 1.1	1.8 ± 1.1	2.1 ± 1.2	1.9 ± 1.2	< 0.001	2.2 ± 1.1	2.3 ± 1.1	2.3 ± 1.2	2.6 ± 1.4	1.7 ± 1.4	0.02
1-year death risk (MAGGIC score)	13 [8–22]	13 [9–23]	13 [8–19]	13 [9–21]	11 [8–18]	0.001	12 [7–19]	14 [8–23]	12 [7–21]	19 [8–27]	8 [4–18]	0.19
3-year death risk (MAGGIC score)	32 [21–49]	32 [23–49]	32 [21–43]	32 [23–46]	27 [19–40]	0.001	29 [19–43]	33 [21–49]	29 [18–46]	43 [21–56]	20 [11–40]	0.10
BMI, kg/m^2¶^	27 [24–31]	27 [24–30]	27 [24–29]	27 [24–30]	27 [24–30]	0.58	28 [24–33]	28 [25–33]	27 [24–31]	27 [24–31]	28 [25–31]	0.52
Obesity^¶^^,c^	122 (29.2)	98 (24.2)	93 (22.4))	97 (25.3)	99 (27.0)	0.57	19 (38.8)	21 (34.4)	16 (28.6)	24 (27.0)	33 (31.4)	0.30
NT-proBNP, ng/L^¥^	-	1675 [713–3709]	1752 [688–4609]	2085 [941–4858]	1595 [720–3630]	0.58	-	785 [233–2010]	1329 [444–3047]	1800 [680–4180]	1007 [254–3709]	0.21
**Treatments**												
ACEI/ARB/ARNI	391 (92.4)	372 (90.3)	379 (90.9)	338 (87.8)	335 (91.3)	0.24	38 (76.0)	48 (77.4)	37 (64.9)	47 (52.8)	55 (51.9)	< 0.001
ARNI	14 (3.3)	26 (6.3)	48 (11.5)	39 (10.1)	125 (34.1)	< 0.001	0 (0.0)	0 (0.0)	0 (0.0)	0 (0.0)	5 (4.7)	0.010
Beta-blocker	351 (83.0)	377 (91.5)	392 (94.0)	368 (95.6)	354 (96.5)	< 0.001	28 (56.0)	43 (69.4)	46 (80.7)	67 (75.3)	78 (73.6)	< 0.05
MRA	247 (58.4)	244 (59.2)	267 (64.0)	295 (76.6)	313 (85.3)	< 0.001	29 (58.0)	27 (43.5)	29 (50.9)	44 (49.4)	39 (36.8)	0.04
Loop diuretic	396 (93.6)	379 (92.0)	372 (89.2)	354 (91.9)	335 (91.3)	0.26	46 (92.0)	59 (95.2)	52 (91.2)	77 (86.5)	63 (59.4)	< 0.001
Digoxin	195 (46.1)	194 (47.1)	157 (37.6)	147 (38.2)	96 (26.2)	< 0.001	25 (50.0)	30 (48.4)	18 (31.6)	29 (32.6)	17 (16.0)	< 0.001
Ivabradine	35 (8.3)	50 (12.1)	107 (25.7)	133 (34.5)	146 (39.8)	< 0.001	1 (2.0)	1 (1.6)	3 (5.3)	5 (5.6)	6 (5.7)	0.15
CRT	35 (8.3)	39 (9.5)	61 (14.6)	61 (15.8)	49 (13.4)	0.001	1 (2.0)	1 (1.6)	1 (1.8)	4 (4.5)	3 (2.8)	0.47
ICD	59 (13.9)	61 (14.8)	81 (19.4)	71 (18.4)	62 (16.9)	0.09	1 (2.0)	0 (0.0)	3 (5.3)	10 (11.2)	8 (7.5)	0.02
Cardiac TR	3 (0.7)	4 (1.0)	2 (0.5)	4 (1.0)	0	0.36	0	0	1 (1.8)	0	0	–

Data are given as mean ± standard deviation, median [interquartile range], or n (%).

*n = 2119; ^#^n = 2083; ^†^n = 1506; ^§^n = 2361; ^‡^n = 2345; ^¶^n = 2350; ^¥^n = 1665.

ACEI, angiotensin-converting enzyme inhibitor; AF/FT, atrial fibrillation or flutter; ARB, angiotensin II receptor blocker; ARNI, angiotensin receptor–neprilysin inhibitor; BMI, body mass index; CM, cardiomyopathy; COPD, chronic obstructive pulmonary disease; Cardiac TR, cardiac transplant; CRT, cardiac resynchronization therapy; HD, heart disease; HF, heart failure; ICD, implantable cardioverter-defibrillator; LVEF, left ventricular ejection fraction; MAGGIC, Meta-analysis Global Group in Chronic Heart Failure; MRA, mineralocorticoid receptor antagonist; NYHA, New York Heart Association; NT-proBNP, N-terminal pro-brain natriuretic peptide.

^a^According to World Health Organization criteria (< 13 g/dL in men and < 12 g/dL in women).

^b^Estimated glomerular filtration rate (Chronic Kidney Disease-Epidemiology Collaboration equation) < 60 mL/min/1.73 m^2^.

^c^Body mass index ≥ 30 kg/m^2^.

### Treatment during follow-up

In the depressed LVEF group, a significant trend towards an increase in several life-saving treatments (beta-blockers, mineralocorticoid receptor antagonists, ivabradine, and CRT) was observed across quintiles, whereas the opposite occurred with digoxin. In patients with preserved LVEF, the use of beta-blockers and ICD (of which, more than a half [54.5%] were implanted in patients with HCM) increased, whereas the use of renin-angiotensin system blockers, loop diuretics, and mineralocorticoid receptor antagonists decreased (Table 2).

### Changes in LVEF at 1 year

LVEF at 1 year of follow-up was available in 1321 out of the 2004 patients with depressed LVEF and in 185 out of the 364 patients with preserved LVEF. LVEF at 1 year increased significantly during the study periods in patients with depressed LVEF (p = 0.001) while it did not change in patients with preserved LVEF (Table 2).

### Mortality trends according to period of admission quintiles

**Figure 1:**
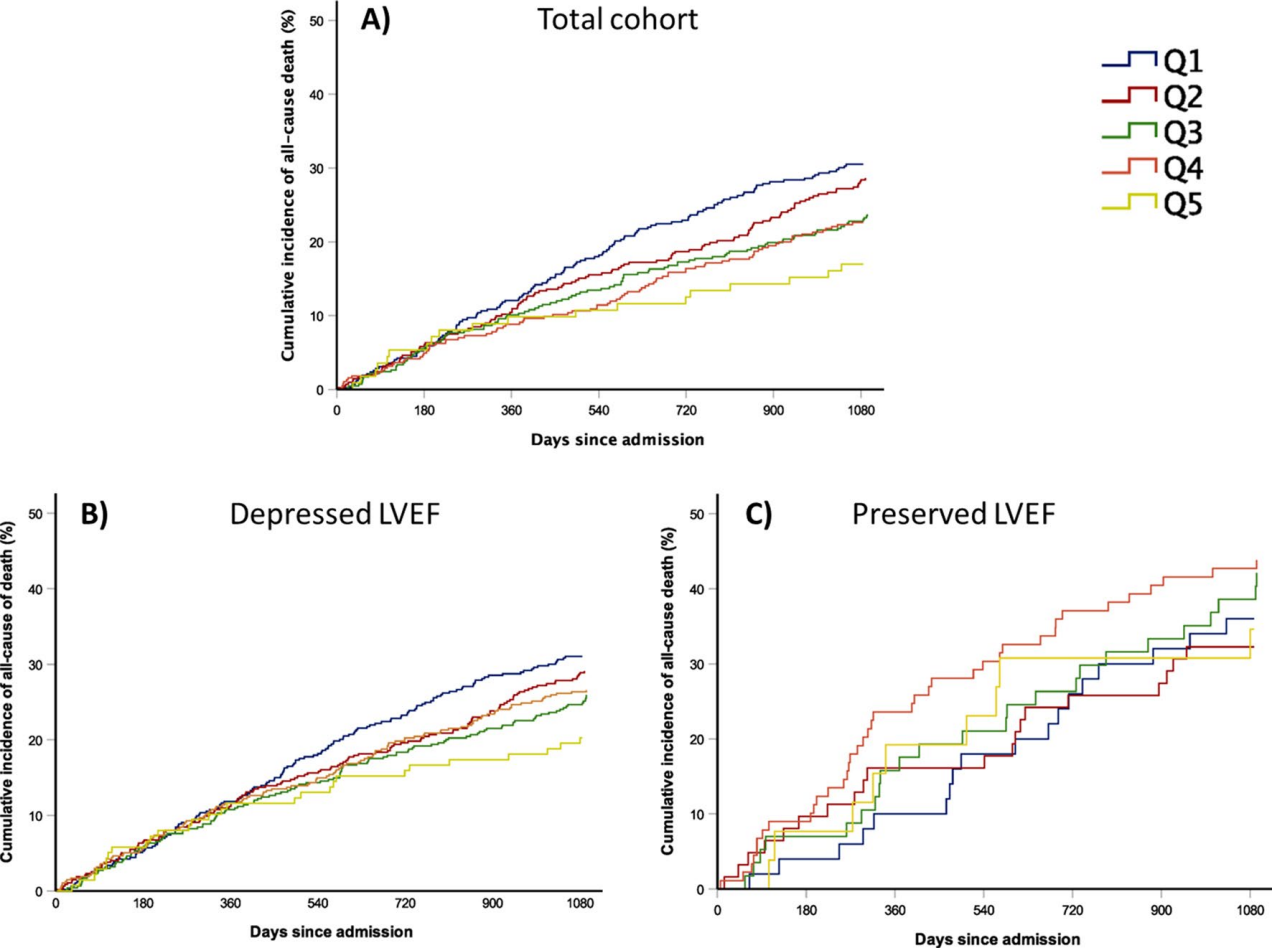
Cumulative incidence of death based on quintiles of period of admission; (**A**) Total cohort population; (**B**) Patients with depressed LVEF; (**C**) Patients with preserved LVEF. Q1, quintile 1, August 2001-August 2004; Q2, quintile 2, September 2004-November 2008; Q3, quintile 3, November 2008-June 2012; Q4, quintile 4, June 2012-October 2015; Q5, quintile 5, October 2015-September 2018.

**Figure 2:**
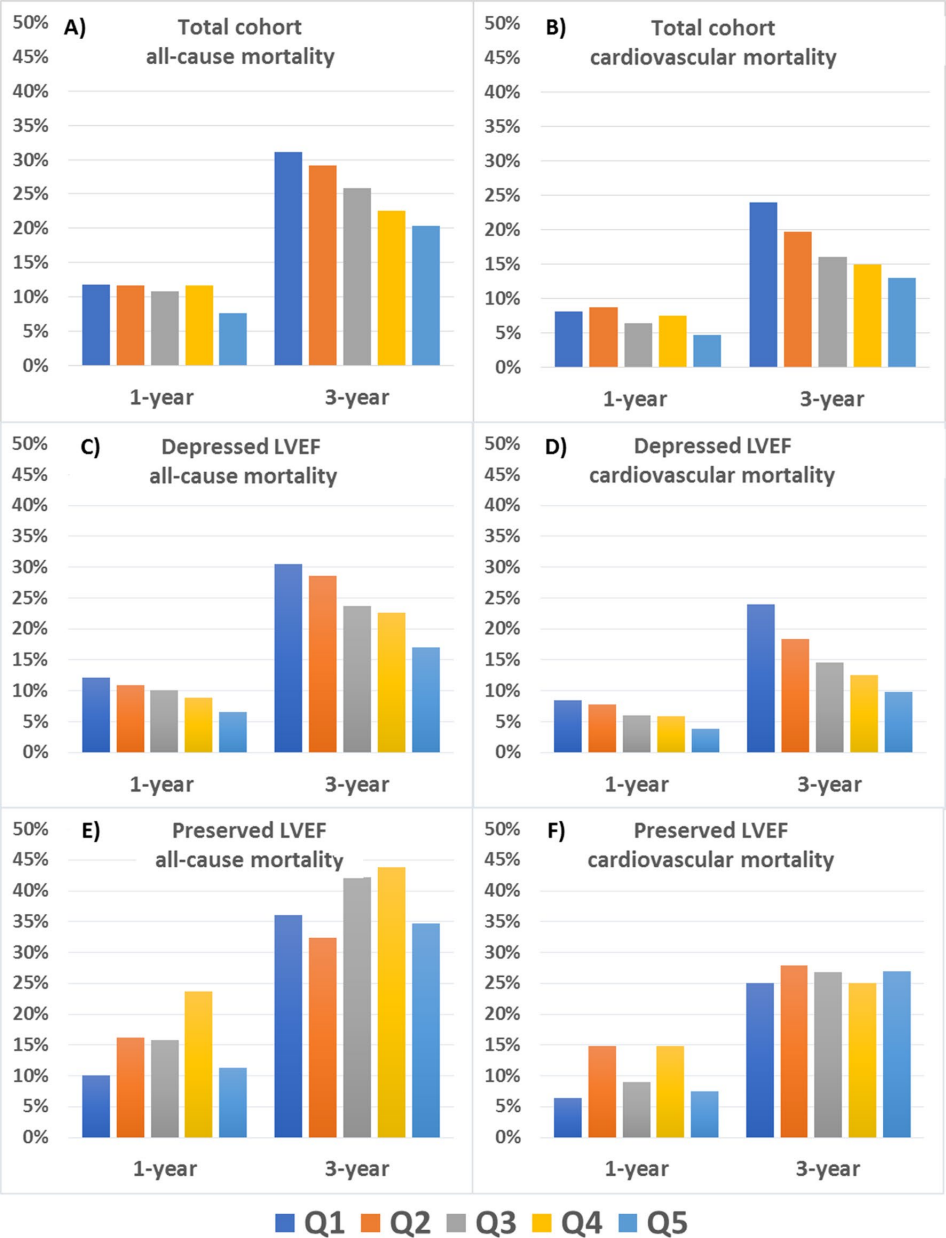
Trends in all-cause and cardiovascular mortality based on quintiles of period of admission. Upper panel, total cohort; middle panel, patients with depressed LVEF; bottom panel, patients with preserved LVEF. (**A**,**C**,**E**) All-cause death; (**B**,**D**,**F**) Cardiovascular death.

During the study period (median follow-up 4.14 years [IQR 1.9–7.7] for the total cohort; 2.9 [IQR 1.4–5.8] for HFpEF patients; and 4.4 [IQR 2.1–8.2] for patients with LVEF < 50%), 1308 patients (55.2%) died, 708 (54.1%) from cardiovascular causes (Supplementary Table S1 online). Supplementary Fig. 1 online shows Kaplan–Meier survival curves for the total cohort and the full follow-up at the HF Unit, according to the period of admission based on quintiles. Mortality was assessed in all patients at 1 year and in 2033 patients at 3 years. Figure 1 shows the cumulative incidence of death according to the period of admission for the total cohort and the two LVEF groups. In the overall HF population, no significant linear trend was observed with regards to all-cause (p = 0.14) and cardiovascular death (p = 0.10) at 1 year, but at 3 years, all-cause death rates fell progressively from 31.1% in Q1 to 20.3% in Q5 (p = 0.02) and cardiovascular mortality decreased significantly from 24.0% in Q1 to 13.0% in Q5 (p = 0.006) (Fig. 2A,B). This linear trend remained significant for all-cause mortality after adjusting the multiple regression models for potential confounding factors (model 1, p = 0.02) and MAGGIC risk score (model 2, p < 0.001; Table 3). Similarly, this trend remained significant for cardiovascular mortality after adjusting for the same confounding factors (model 1, p = 0.006; Table 3) and MAGGIC risk score (model 2, p = 0.04; Table 3). As depicted in Fig. 2C,D, in patients with depressed LVEF, we found a progressive decrease in both all-cause and cardiovascular death rates at 1 year (from 12.1% in Q1 to 6.5% in Q5, p = 0.003; and from 8.4% in Q1 to 3.8% in Q5, p = 0.007, respectively); and 3 years (from 30.5% in Q1 to 17.0% in Q5, p = 0.003; and from 23.9% in Q1 to 9.8% in Q5, p = 0.003, respectively). Almost all of these trends remained significant after adjusting for confounding factors and risk: all-cause mortality at 1 year (model 1, p = 0.003; model 2, p = 0.04) and 3 years (model 1, p = 0.003; model 2, p = 0.03), and cardiovascular mortality at 1 year (model 1, p = 0.007; model 2, p = 0.05) and 3 years (model 1, p = 0.003; model 2, p = 0.02; Table 3). Conversely, in the preserved LVEF subgroup, all-cause mortality rates at 1 and 3 years ranged from 10.0% to 11.3% and from 36.0% to 34.6%, with no significant trend over time (p = 0.63 and p = 0.65, respectively; Fig. 2E–F). In the same subgroup of patients, we observed no significant trends in terms of cardiovascular mortality at 1 (p = 0.88) or 3 years (p = 0.86). In a sensitivity analysis, mortality was also explored using a preserved LVEF threshold of ≥ 40%. Again, we did not find any significant trend in all-cause death (at 1 year, p = 0.41; at 3 years, p = 0.98) nor in cardiovascular mortality (at 1 year, p = 0.45; at 3 years, p = 0.93).

**Table 3:** Data for periods of admission in multiple regression analyses.

	Beta-value	Standard error	95% CI	p-value
**Model 1**				
Total cohort (n = 2368)				
All-cause death				
3 years	− 2.410	0.535	− 4.114, − 0.706	0.02
Cardiovascular death				
3 years	− 2.680	0.388	− 3.916, − 1.444	0.006
LVEF < 50% (n = 2004)				
All-cause death				
1 year	− 1.330	0.155	− 1.823, − 0.837	0.003
3 years	− 3.300	0.383	− 4.520, − 2.080	0.003
Cardiovascular death				
1 year	− 1.110	0.164	− 1.632, − 0.588	0.007
3 years	− 3.410	0.394	− 4.665, − 2.155	0.003
**Model 2**				
Total cohort (n = 2368)				
All-cause death				
3 years	− 1.906	0.026	− 2.020, − 1.792	< 0.001
Cardiovascular death				
3 years	− 2.719	0.538	− 5.035, − 0.402	0.04
LVEF < 50% (n = 2004)				
All-cause death				
1 year	− 1.070	0.066	− 1.352, − 0.788	0.004
3 years	− 2.860	0.497	− 4.999, − 0.721	0.03
Cardiovascular death				
1 year	− 0.930	0.220	− 1.876, 0.016	0.05
3 years	− 3.810	0.554	− 6.193, − 1.427	0.02

**Model 1**: Quintiles of admission period, age, sex, left ventricle ejection fraction, NYHA class III-IV, and number of comorbidities (diabetes, hypertension, anaemia, atrial fibrillation or flutter, chronic obstructive pulmonary disease). Multiple regression was stepwise analysis.

**Model 2**: Quintiles of admission period and MAGGIC risk score at 1 and 3 years.

### HF hospitalizations according to period of admission quintiles

No trend in the HF hospitalizations was found at 1 year (p = 0.79) nor at 3 years (p = 0.76) in the total cohort, although in those patients admitted in the last quintile period there were significantly less hospital admissions than in the other groups. The same figure was observed both for patients with depressed (p = 0.63 and 0.53, respectively) and for patients with preserved LVEF (p = 0.90 and 0.90, respectively) (Supplementary Fig. 2).

## Discussion

The main finding of our study is that mortality rates (both all-cause and cardiovascular) declined progressively at 3 years in a real-life ambulatory HF population over a 19-year period. In addition, patients with depressed LVEF had a significant reduction in all-cause and cardiovascular mortality at 1 and 3 years, but no significant trend in mortality was observed in patients with preserved LVEF.

Over the past 30 years, the survival of patients with HF has improved remarkably, in particular across trials including patients with HFrEF. Due to the introduction of drugs that inhibit the neurohormonal system and devices such as ICD and CRT, 1-year mortality rates in HFrEF patients have declined from approximately 15% in the Studies of Left Ventricular Dysfunction (SOLVD) trial in 1991 to less than 10% in the recent Prospective Comparison of ARNI with ACEI to Determine Impact on Global Mortality and Morbidity in Heart Failure (PARADIGM-HF) and Dapagliflozin and Prevention of Adverse-outcomes in Heart Failure (DAPA-HF) trials^1–10^. However, patients included in randomized clinical trials (RCTs) usually differ from those that physicians manage every day in clinical practice; they are usually younger, more stable, have fewer comorbidities, higher adherence rates to therapy, and attend more follow-up visits than real-world patients^17,18^. In a recent systematic review of 118 trials published between 2001 and 2016, Khan et al. found that 72% excluded patients with ≥ 1 comorbid condition among dementia, anaemia, diabetes mellitus, severe or uncontrolled hypertension, chronic kidney disease (CKD), atrial fibrillation, chronic liver disease, stroke, cancer, and COPD^19^. CKD was the most common exclusion criterion (47% of trials). Regarding real-world data, in an Italian population-based database including 41,413 patients discharged from the hospital with a diagnosis of HF, the mean age was 78 ± 11 years and comorbidities, such as COPD, CKD, or cancer, accounted for up to 30% of cases^20^. Furthermore, recent data from 207,984 patients in the Get-With-The Guidelines-Heart Failure-registry (GWTG-HF) indicate that the prevalence of 0, 1, 2, and ≥ 3 non-cardiovascular comorbidities is 18%, 30%, 27%, and 25%, respectively^21^. Another important issue that needs to be addressed is the significant gap in the use of guideline-directed medical therapy between RCTs and contemporary HF registries. As shown in the recent Change-the Management-of-Patients-with-Heart-Failure (CHAMP-HF) and the Chronisch-Hartfalen-ESC-richtlijn-Cardiologische-praktijk Kwaliteitsproject-HartFalen (CHECK-HF) registries, ACEI/ARB/ARNI, beta blockers and MRA were prescribed in up to 84%, 86% and 56% of eligible patients with HFrEF, respectively^22,23^.

These differences between RCTs and registries translate into an actual worse prognosis for real-world HF patients. A study from two U.S. community-based samples that included patients with HF from 1990 to 2009 reported that 67.4% of patients died at 5 years, and that mortality rates did not improve significantly across the decades considered^24^. Prognostic data from the GWTG-HF registry revealed that mortality in hospitalized HF patients can be as high as 75% at the 5-year follow-up, regardless of the LVEF^12^.

Despite being relatively young (66.4 ± 12.9 years), patients admitted to our HF clinic had 1.9 ± 1.2 comorbidities (among diabetes, hypertension, anaemia, atrial fibrillation or flutter, COPD), and the overall prevalence of COPD, CKD, and anaemia was 17.1%, 44.1%, and 45.4%, respectively. With regards to prognosis, at the beginning of the study period (2001–2004), mortality rates were between those reported by RCTs and the rates from registries (11.8% at 1 year and 31.1% at 3 years for all-cause death; 8.1% at 1 year and 24.0% at 3 years for cardiovascular death). Nevertheless, by 2015–2018, mortality rates were similar to those from contemporary RCTs, especially in the depressed LVEF subgroup (6.5% at 1 year and 17% at 3 years for all-cause death; 3.8% at 1 year and 9.8% at 3 years for cardiovascular death). This could be related to the fact that our patients were progressively treated according to the trials demonstrating benefits of disease-modifying drugs and devices. In fact, in patients with LVEF < 50%, we found a significant increase in the prescription rates for neurohormonal antagonists, which reached 91.3% for ACEIs, ARBs, or ARNIs, 96.5% for beta-blockers, and 85% for MRAs in the last quintile. Similarly, ICD and CRT rates increased significantly by 2015–2018, to 16.9% and 13.4%, respectively. On the other hand, patients with depressed LVEF admitted more recently reported less severe NYHA class symptoms, had higher LVEF, and lower MAGGIC risk score. Nevertheless, and very remarkable, the trend of lower mortality in these patients remained significant after adjusting for clinical confounders, such as age, sex, NYHA class, comorbidities, and LVEF, as well as the risk estimated by the MAGGIC score.

With respect to patients with preserved LVEF, survival did not improve over the last two decades in our cohort. Although a recent meta-analysis supports a possible beneficial effect of neurohormonal inhibitors in HFpEF^25^, no RCT has yet demonstrated a clear impact of any pharmacological treatment on survival in patients with HFpEF^11^. HFpEF is a very heterogeneous entity, and some authors have suggested phenomapping patients in order to classify them into different groups^26^. Personalized treatment based on this classification has been proposed^27^, but to the best of our knowledge improved prognosis has not been demonstrated. In addition, patients with HFpEF admitted to our unit were older (69.1 ± 14.5 vs. 65.9 ± 12.5, p < 0.001) and had more comorbidities (2.2 ± 1.3 vs. 1.8 ± 1.2, p < 0.001) than the depressed LVEF subgroup, which indicates that the condition would be more difficult to influence with cardiac treatment. Nevertheless, patient age and comorbidities decreased significantly over time in our cohort, though no mortality reduction was observed. Finally, a recent review based on current evidence suggests that disease management programs for HF may improve survival and other outcomes in HFpEF patients, given that they are older and multi-morbid, and their management should not rely on a single-disease focus, but provide comprehensive care after geriatric assessment^28^. Our HF Unit is multidisciplinary and addresses the non-cardiac issues of HF patients, including comorbidities, geriatric evaluation, and rehabilitation. Despite this, no improvement in survival has been observed over the last two decades in patients with preserved LVEF. However, only one of the revised studies was focused on HFpEF, and it did not reduce all-cause or cardiovascular mortality^29^.

## Study limitations

Our study has some limitations. First, the study cohort was a general HF population treated at a specific multidisciplinary HF clinic in a tertiary care hospital, with most patients referred from the Cardiology Department; thus, there was a predominance of relatively young men with HF of ischaemic aetiology and depressed LVEF, and the population was almost exclusively White. Therefore, we may not be able to fully extrapolate the results to other populations. Notably, a common treatment protocol was applied to all patients, limiting the possible bias introduced by different management strategies or treatment protocols. Second, the limited number of patients with preserved LVEF in our cohort makes the analysis of this subgroup less robust.

## Conclusion

In a cohort of real-life ambulatory patients with HF of different aetiologies attended at a specialized HF clinic in a tertiary centre, mortality has progressively declined in patients with LVEF < 50%, but the same trend has not been observed in patients with preserved LVEF.

## Supplementary Information

Supplementary Information 1
