# Supplementary material for: Mortality trends in an ambulatory multidisciplinary heart failure unit from 2001 to 2018
Source: Sci Rep. 2021 Jan 12;11:732. doi: 10.1038/s41598-020-79926-3 (PMC7804393; doi:10.1038/s41598-020-79926-3)
Supplement: Supplementary file 1 — Supplementary Information 1 [file 41598_2020_79926_MOESM1_ESM.docx]

**Mortality Trends in an Ambulatory Multidisciplinary Heart Failure Unit from 2001 to 2018**

**^SUPPLEMENTARY MATERIAL^**

Giosafat Spitaleri, MD^a^; Josep Lupón, MD, PhD^a,b,c^; Mar Domingo, MD, PhD^a^; Evelyn Santiago-Vacas, MD^a^; Pau Codina, MD^a^; Elisabet Zamora, MD, PhD^a,b,c^; Germán Cediel, MD, PhD^a,b^; Javier Santesmases, MD^a,b^; Crisanto Diez-Quevedo; MD, PhD^a^, Maria Isabel Troya; MD, PhD^a^, Maria Boldo, MD^a^; Salvador Altmir, MD^a^; Nuria Alonso, MD, PhD^a^; Beatriz González, can; Julio Núñez, MD, PhD^c,d,e^; Antoni Bayes-Genis, MD, PhD^a,b,c^

^a^Heart Failure Clinic and Cardiology Service, University Hospital Germans Trias i Pujol, Badalona, Spain.

^b^Department of Medicine, Universitat Autonoma de Barcelona, Barcelona, Spain.

^c^CIBERCV, Instituto de Salud Carlos III, Madrid, Spain.

^d^Cardiology Department, Hospital Clínico Universitario, INCLIVA, València, Spain.

^e^Department of Medicine, Universitat de València, València, Spain

**Supplementary Table S1.** Causes of death.

| **Cause of death** | **Number** | **Percentage**  **within deaths** | **Percentage**  **of population** |
| --- | --- | --- | --- |
| Unknown | 88 | (6.9) | (3.7) |
| Heart failure | 367 | (28.9) | (15.5) |
| Sudden death | 165 | (13.0) | (7.0) |
| Myocardial infarction | 62 | (4.9) | (2.6) |
| Stroke | 25 | (2.0) | (1.1) |
| Cardiovascular procedural | 17 | (1.3) | (0.7) |
| Other cardiovascular | 72 | (5.7) | (3.0) |
| Non-cardiovascular | 476 | (37.4) | (20.1) |

**Supplementary Figure 1**

**
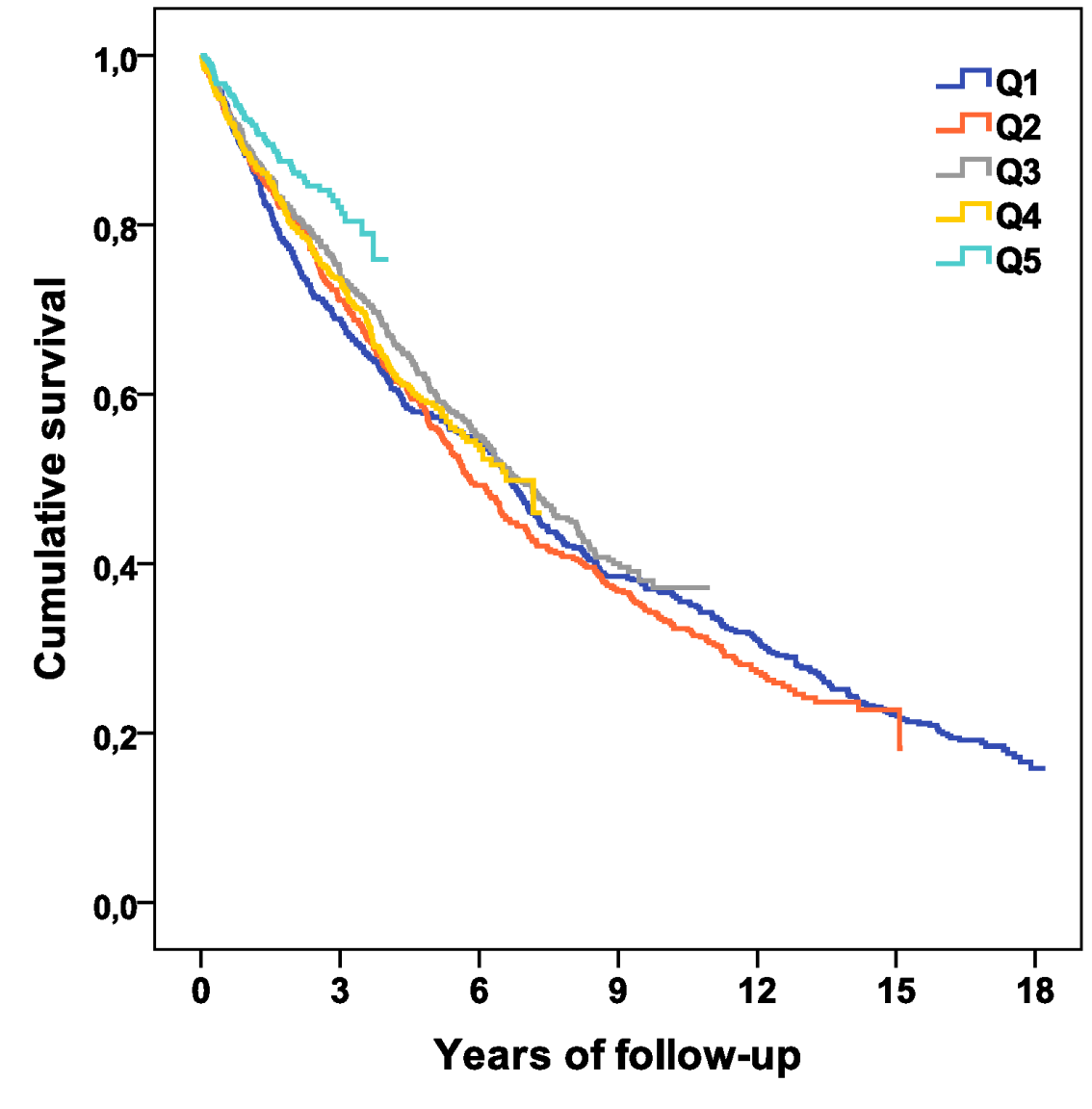
**

**Title.** Kaplan-Meier survival curves based on quintiles of period of admission at the Heart Failure Unit (full follow-up).

**Caption.** Q1, quintile 1, August 2001-August 2004; Q2, quintile 2, September 2004-November 2008; Q3, quintile 3, November 2008-June 2012; Q4, quintile 4, June 2012-October 2015; Q5, quintile 5, October 2015-September 2018.

**Supplementary Figure 2**

**
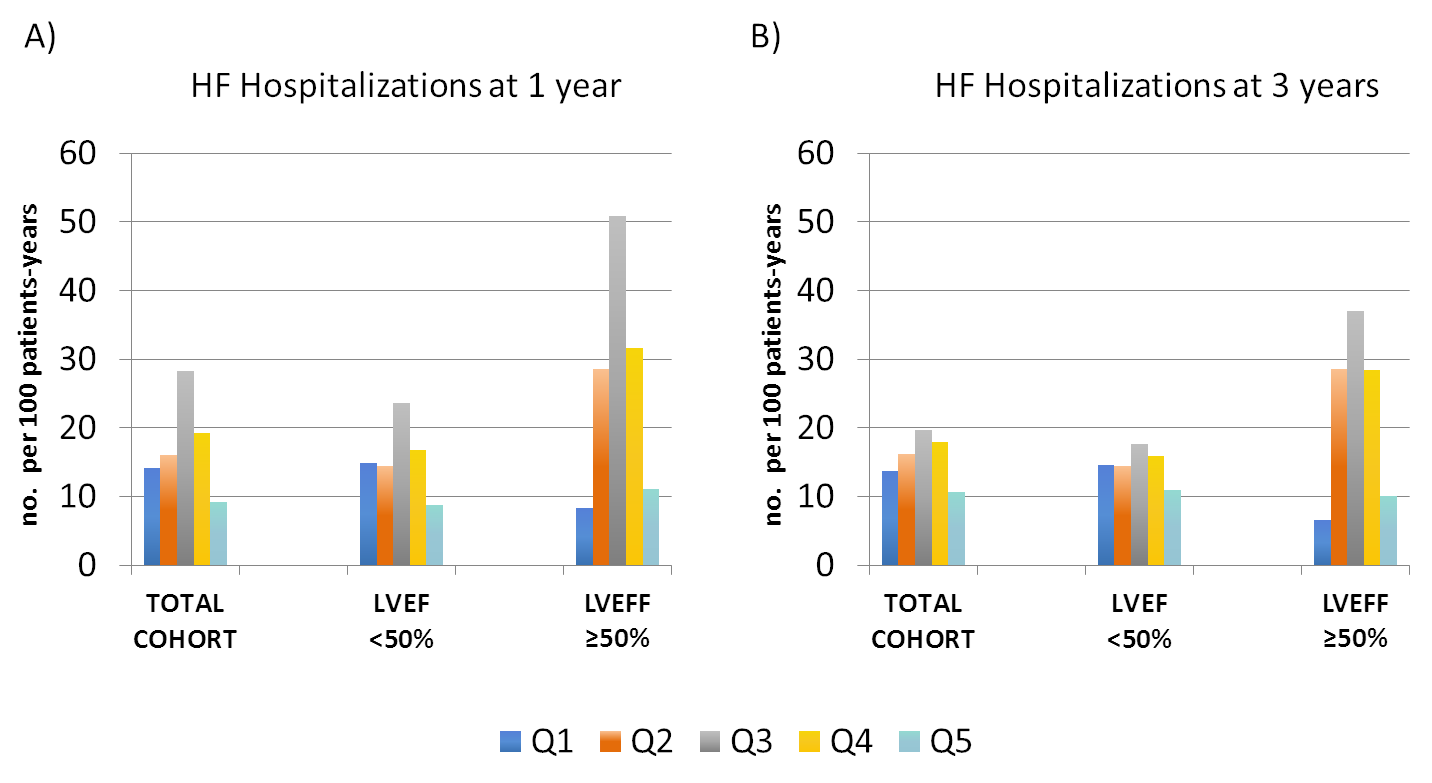
**

**Title.** Recurrent HF-related hospitalizations based on quintiles of period of admission at the Heart Failure Unit

**Caption.** Panel A) HF hospitalizations at 1 year. Panel B) HF hospitalizations at 3 years.

Q1, quintile 1, August 2001-August 2004; Q2, quintile 2, September 2004-November 2008; Q3, quintile 3, November 2008-June 2012; Q4, quintile 4, June 2012-October 2015; Q5, quintile 5, October 2015-September 2018.
